# Supplementary material for: Health anxiety, coping mechanisms and COVID 19: An Indian community sample at week 1 of lockdown
Source: PLoS One. 2021 Apr 21;16(4):e0250336. doi: 10.1371/journal.pone.0250336 (PMC8059846; doi:10.1371/journal.pone.0250336)
Supplement: Attachment — Submitted filename: Response to Reviewers .docx [file pone.0250336.s003.docx]

To

The Academic Editor and Reviewers

We would like to thank you for your comments. These are addressed in an itemised manner below:

Academic Editor’s comment

Comment No 1: Journal Requirements:

Please review your reference list to ensure that it is complete and correct. If you have cited papers that have been retracted, please include the rationale for doing so in the manuscript text or remove these references and replace them with relevant current references. Any changes to the reference list should be mentioned in the rebuttal letter that accompanies your revised manuscript. If you need to cite a retracted article, indicate the article’s retracted status in the References list and also include a citation and full reference for the retraction notice.

*Authors’ response No 1: We would like to thank the academic editor for drawing to our attention the reference section. The appropriate formatting has now been used and references checked as advised.*

Reviewers' comments:

Reviewer #1:

 Comment No 2: I’d like to commend the authors for the significant amount of work they clearly put into this revision. They have addressed many of the comments, questions, and issues that the reviewers previously raised.

Although I continue to think this manuscript could make for a useful contribution and I recognize the effort put into more clearly articulating a theoretical model underlying the analysis (an issue I raised in my review of the original document), I do have a small number of questions and comments that I’d like them to consider:

*Authors’ response No 2: We would like to thank the reviewer for their appreciative comments*

Comment No 3: The authors have reported that the fit of their original conceptual model to the data is satisfactory. That’s great and the scores look encouraging, but I wonder if they considered making any revisions to their original model, based on their initial SEM analyses and modification indices. If not, why not? If so, but they ultimately decided to stick with their original model, why was that the case? If they did make changes, what were those?

*Authors’ Response No 3: We would like to thank the reviewer for the opportunity to provide additional information about the models as informed by the SEM analyses. The manuscript on page 11 lines 193-200 now read as: Information RMSEA, S_RMR, CD, TLI and CFI of the individual measures are provided in Table 3 (Model I), demonstrating that the predicted model is an acceptable fit (Byrne, 2016; Kline, 2016).*

*Table 3 Fit Indices of the whole model*

|  |  |  |  | *Fit Index* | | |  |
| --- | --- | --- | --- | --- | --- | --- | --- |
|  | $\chi2$ | *df* | *RMSEA* | *S-RMR* | *CD* | *TLI* | *CFI* |
| *Model I* | *429.934* | *225* | *0.063* | *0.08* | *0.920* | *0.812* | *0.833* |
| *Model II* | *194.749* | *194* | *0.004* | *0.055* | *0.926* | *0.999* | *0.999* |

*It was considered whether any revisions to this model should be made given the acceptable fit indices. Modification indices recommended the inclusion of some disturbance covariance terms between item variables. This resulted in a better data to model fit (Model II) and confirmed our confidence in the structure.*

Comment No 4: It would be helpful to include tables containing the regression weights of the hypothesized theoretical model, for both direct and indirect effect scores. A discussion of possible indirect effects would be helpful, too.

*Authors’ response No 4: The following details have been added to the manuscript on page 12 lines 207-211: ‘There are significant indirect effects mediated by health anxiety from internal and external locus of control to all of the coping strategies as illustrated in the table above. This indicates that a person’s internal and external locus of control have an effect on how they cope.*

*Table 4 Regression standardized structural coefficients*

|  | *Endogenous variable* | | | |
| --- | --- | --- | --- | --- |
| *Exogenous variable* |  |  |  |  |
|  | *Health Anxiety* | *Behavioural Coping* | *Mindful Coping* | *Coping through Social Media* |
| *Internal LoC* |  |  |  |  |
| *Direct effect* | *0.304^***^* |  |  |  |
| *Indirect effect* |  | *0.102^*^* | *0.067^*^* | *0.146^**^* |
|  |  |  |  |  |
| *External LoC* |  |  |  |  |
| *Direct effect* | *0.247^***^* |  |  |  |
| *Indirect effect* |  | *0.083^*^* | *0.054^*^* | *0.119^*^* |
|  |  |  |  |  |
| *Health Anxiety* |  |  |  |  |
| *Direct effect* |  | *0.336^***^* | *0.220^**^* | *0.480^***^* |
|  |  |  |  |  |

*p^***^<0.001, p^**^<0.01, p^*^<0.05. Measurement path coefficients all sig p<0.05*

Comment No 5: Three different age groups are compared. However, it seems to me that a proper multigroup SEM analysis is required to ascertain if the hypothesized structure among the key variables in the theoretical model holds across these groups (or not). Findings in either direction could be useful. At the moment it seems (and please correct me if I am wrong) that there was no formal analysis was done to test differences across age groups.

*Authors’ response No 5: We have added the following details on pages 13-14 lines 232-239: ‘A test for measurement invariance was undertaken on age subgroups comparing the unconstrained model with all parameters free across groups, to one with coefficients invariant. The chi-square and fit indices difference (Δχ^2^(14) = 10.942, p=0.691; ΔRMSEA=0.036; ΔCFI=0.027) demonstrates the models measurement invariance (Rutkowski, & Svetina, 2014; Chen, 2007). The equality of the model was also assessed through metric invariance tests. These tests of the structural model gave non-significant results across all structural path parameters indicating that the constructs within this model hold across groups (Cheung & Rensvold, 2002; Gregorich, 2006).’*

Reviewer #2:

Comment No 6: Thank you for the opportunity to re-review this manuscript. All initial reviewer comments have been addressed satisfactorily by the authors.

A final clarification remains regarding Author Response 32-

Has correct methodology been utilized to adapt and validate the study instruments to the Indian cultural context? If so, please cite them within the article. If not, clearly state the failure to use any specific guidelines within the limitations of the study. At present, the limitations note that several scales were adapted, however, not “fully” validated—a potential misrepresentation as adherence to any recognized method or guidelines for cross-cultural adaptation and validation is not observed within study.

*Authors’ response No 6: We thank the reviewer for both appreciating the responses and drawing our attention to the above. The limitations section on page 16 lines 303-305 now reads as: ‘A number of scales were adapted for this study. Although the internal consistency for these measures is encouraging, cross cultural adaptation and validation is yet to be undertaken.’*
